# The Role of Empathy in Health and Social Care Professionals

**DOI:** 10.3390/healthcare8010026

**Published:** 2020-01-30

**Authors:** Maria Moudatsou, Areti Stavropoulou, Anastas Philalithis, Sofia Koukouli

**Affiliations:** 1 Department of Social Work, Hellenic Mediterranean University, 71410 Heraklion, Greece; moudatsoum@yahoo.gr; 2 Laboratory of Interdisciplinary Approaches for the Enhancement of Quality of Life (Quality of Life Lab), Hellenic Mediterranean University, 71410 Heraklion, Greece; aretistavropoulou@gmail.com; 3 Centre of Mental Health, 71201 Heraklion, Greece; 4 Nursing Department, University of West Attica, 12243 Athens, Greece; 5 Department of Social Medicine, Medical School, University of Crete, 70013 Heraklion, Greece; philal@uoc.gr

**Keywords:** empathy, health care professionals, social care professionals, therapeutic relationship, communication, health care users

## Abstract

The current article is an integrative and analytical literature review on the concept and meaning of empathy in health and social care professionals. Empathy, i.e., the ability to understand the personal experience of the patient without bonding with them, constitutes an important communication skill for a health professional, one that includes three dimensions: the emotional, cognitive, and behavioral. It has been proven that health professionals with high levels of empathy operate more efficiently as to the fulfillment of their role in eliciting therapeutic change. The empathetic professional comprehends the needs of the health care users, as the latter feel safe to express the thoughts and problems that concern them. Although the importance of empathy is undeniable, a significantly high percentage of health professionals seem to find it difficult to adopt a model of empathetic communication in their everyday practice. Some of the factors that negatively influence the development of empathy are the high number of patients that professionals have to manage, the lack of adequate time, the focus on therapy within the existing academic culture, but also the lack of education in empathy. Developing empathetic skills should not only be the underlying objective in the teaching process of health and social care undergraduate students, but also the subject of the lifelong and continuous education of professionals.

## 1. Introduction

Communication skills have been described as the most important ability for a health professional. Efficient communication depends upon the therapist feeling certain that they have really heard and recorded the health care user’s needs so as to provide personalized care [1]. It is important for health professionals to understand people’s feelings, opinions and experiences in order to assess their real needs and act accordingly, offering tailor-made services. Reaching that goal makes the development of empathetic skills necessary [2].

The concept of empathy is a common denominator for many health professionals such as nurses, doctors, psychologists, and social workers [3,4,5,6]. The person-centered approach for the unconditional acceptance of the health care user and empathy have for years been the fundamental values in the education and implementation of clinical social practice [3,7,8,9]. 

## 2. Material and Methods 

The aim of the present paper was to analyze the concept of empathy and emphasize its importance to the health professions. The research questions under consideration have been the following: 1. What does empathy mean and which are its dimensions; 2. What are the role and meaning of empathy in health and social care professions for the therapeutic journey of the health care user; 3. How can we assess the levels of empathy in professionals (assessment tools); 4. Which factors influence empathy?

A literature search was conducted by searching PubMed and Scopus databases, to identify studies of the last fifteen years published in English and Greek language. The key-words used were ‘empathy’ and ‘health professionals’. Out of the search, 78 studies were identified that better answer the aim and purposes of the present paper. These studies were discussed and evaluated by the authoring team in order to reach consensus on the eligibility of each one with the proposed research questions. After agreement was reached, re-examination and analysis of the studies’ findings lead to the formulation of four thematic categories, namely, a) Concept definition and dimensions, b) The role of empathy in health and social care professionals, c) Assessing empathy, and d) Factors that influence empathy.

## 3. Results

### 3.1. Concept Definition and Dimensions

Empathy is the ability to understand and share other people’s feelings [10]. It is a core concept as, according to the psychodynamic, behavioral and person-centered approaches, it facilitates the development of a therapeutic relationship with the health care user, providing the basis for therapeutic change [11].

Empathy was first mentioned in a psychotherapeutic context in the 1950s [7]. The person-centered approach defined it as the temporary condition that a health professional experiences in his/her effort to understand a health care user’s life without bonding with them [3,12].

The contemporary concept of empathy is multidimensional and consists of affective, cognitive, and behavioral aspects [6,11,13]. Throughout history, the development and integration of this concept evolved along three different time periods. Until the end of the 1950s, the cognitive dimension was mostly prevalent. From 1960 onwards, emphasis was given to the affective dimension, whereas since 1970, empathy has been defined in all its multi-dimensionality; that is, the behavioral aspect has been added to the everyday practice of the health care professionals [14].

The affective dimension consists of the concepts of caring and that of the sincere, unconditional acceptance of the health care user (congruence) [8,15,16]. Caring refers to the assistance and support as byproducts of an emotional interaction. The concept of the full and sincere unconditional acceptance refers to the approval of the ‘other’ and a consensus between people, without preconceptions or stereotypes. 

The cognitive dimension pertains to the interpersonal sensitivity and the ability to understand the position the other person is in (perspective taking) [17,18]. Interpersonal sensitivity means objectively understanding the other person’s situation. It is a deep process of getting to know someone, based in both verbal and non-verbal cues. The ability to understand the other person’s situation refers to the flexibility and the objective understanding of the point of view of the other person (walk in their shoes, comprehending the way they perform cognitively, emotionally, and mentally) [17,18].

Altruism and the therapeutic relationship both belong to the behavioral dimension which develops empathy into practice [19,20]. Altruism is a socially directed behavior aimed at relieving difficulties, problems, and the pain associated with them [11].

Sympathy, empathy, and compassion are closely related terms that are often used interchangeably. Sympathy has been defined as an emotional reaction of pity toward the misfortune of another, especially those who are perceived as suffering unfairly [21]. Empathy is understood as a more complex interpersonal construct that involves awareness and intuition, while compassion is a ‘complementary social emotion, elicited by witnessing the suffering of others’ and is related with the feelings of concern, warmth associated to motivating of support [22]. Empathetic listening might result in compassion fatigue because of prolonged exposure to stress and all it evokes [23]. Self-care practice, well-being, and self-awareness are fundamental in enhancing empathy and reducing compassion fatigue [23,24].

### 3.2. The Role of Empathy in Health and Social Care Professionals

In a qualitative research study, nurse students, who were asked their opinion on empathy, emphasized the three dimensions of the concept [3]. Participants described it as the nurse’s ability to understand and experience other people’s feelings, thoughts, and wishes, as well as the nurse’s capacity to comprehend the emotional and cognitive state of the person they work with. To sum up, empathy is perceived as a combination of the emotional, cognitive and practical skills involved when caring for a patient [3].

Empathy is one of the fundamental tools of the therapeutic relationship between the carers and their patients and it has been proven that its contribution is vital to better health outcomes [8,25,26]. As it allows the health care providers to detect and recognize the users’ experiences, worries, and perspectives [27], it strengthens the development and improvement of the therapeutic relationship between the two parts [28]. It is widely acknowledged that the health professional’s empathetic ability leads to better therapeutic results [29].

The empathetic relationship of the health professionals with their health care users reinforces their cooperation towards designing a therapeutic plan and a tailor-made intervention, increasing thus the patient’s satisfaction from the therapeutic process. This way, quality of care is enhanced, errors are eliminated, and an increased percentage of health care recipients positively experience therapy [30,31,32,33,34]. Furthermore, it has been noted that the empathetic relationship developed during the process of care reinforces the therapeutic results, as the users better comply with the therapeutic course of action [34].

Studies performed in various groups of patients with different health problems generated positive results regarding the progress of their health. Specifically, studies of patients with diabetes showed that there is an association between empathy and the positive therapeutic course of disease [31,35]. Moreover, patients with cancer demonstrate less stress, depression, and aggressiveness when receiving empathetic nursing care [36]. The empathetic relationship between a midwife and a future mother increases the latter’s satisfaction and lessens the stress, the agony, and the pain of the forthcoming labor as the mother feels security, trust, and encouragement [37].

Understanding based on empathy is critical to the relationship between the health professional and the recipient of care. When that happens, health care users feel secure and trust the professional’s abilities. Therefore, the distance between the expert and the patient shortens and both of them come closer, enjoying mutual benefits [12]. Moreover, a relationship based on empathy helps the therapists lessen their stress and burnout in the workplace and adds to their quality of life [37,38]. It has been shown that physicians who have higher levels of empathy experience less burnout or depression [39,40].

Empathy is especially important to the social care professions. It has been noted that the ability of the social worker for empathy and understanding of the users’ experiences and feelings plays a crucial role in social care as empathy is one of the most important skills that these professionals may employ to develop a therapeutic relationship [5,41].

Health care users who experience empathy during their treatment exhibit better results and a higher possibility for a potential improvement [42]. Moreover, social workers with higher levels of empathy work more efficiently and productively as to the fulfillment of their role in creating social change [13]. This happens because empathy helps the social worker understand and feel compassion towards their health care users so as the latter can feel secure to express their thoughts and problems. This way, a basis for trust is created, one that leads to therapeutic change and the improvement of the care recipient’s overall social functionality [13]. Social functionality levels are assessed by the social worker and refer to the ability of a person to accomplish their everyday activities (preparing and keeping meals, seeking accommodation, taking care of their selves, commuting) as well as their ability to fulfill social roles (parent, employee, member of a community) according to the requirements of their cultural environment [43].

Empathy contributes to the precise assessment of the situation the health care user is in. It offers the therapists the chance to make good use of non-verbal cues (behavior modeling, body movements, tone of voice, etc.) and helps them manage the user’s emotions. What is more, empathy enhances the user’s ability to comprehend reality and improve the quality of their life [13].

### 3.3. Assessing Empathy 

Although both health care users and health professionals consider empathy as very important for the development of the therapeutic relationship and a necessary skill for a therapist, studies show a reduction of empathy in professional relationships. Often, health care users believe that health professionals do not understand the situation that health care users are in, whereas research findings showed that health professionals and health care users have different views on the communication abilities of the former, as if they come from different worlds [44,45]. It is especially important that—according to research findings deriving from medical student samples—empathy seems to increase in the first year of studies, but starts decreasing around the third year and remains low up to graduation [46,47].

As mentioned before, there are different dimensions, but also levels of empathy. Accordingly, there are different assessment scales for professionals and patient-users [48]. 

One of the most important tools for the quantitative assessment of empathy is the Jefferson Scale of Empathy (JSE) which was originally used to evaluate empathy in medical students [27,49]. Subsequently, its use was extended to other professional groups also, for example physicians, health professionals in general and students of other health professions [27,49,50,51]. The Jefferson scale has been used in many countries, such as the USA, Poland, Korea, Italy, Japan and has been standardized for its validity and reliability [12,49,50,52,53]. It is self-administered and completed by physicians and other health professionals who provide care to patients in clinical settings. Moreover, students of medical, nursing, and other health care sciences may also complete it. The scale includes 20 questions and the overall score ranges from twenty to one hundred and forty; higher scores indicate a better empathic relationship in the medical and therapeutic care [26,49,53,54].

More specifically, for social work, the Empathy Scale for Social Workers (ESSW) is a questionnaire designed for the quantitative assessment of empathy in social care professionals and students. It can be very useful in practice settings to support decision making processes, assist career choice decisions, continuing education, and supervision needs in the field of social care. Its usefulness is also underscored for potential social work supervisors, as it helps identifying the types of empathy needed while supervising clinicians and staff. The scale is a screening and self-evaluation tool completed by social work students and practitioners [13]. It consists of 41 questions and every question is marked on a five point scale and higher scores indicate higher levels of empathy [13]. 

### 3.4. Factors that Influence Empathy 

As mentioned before, although research has showed the value of empathy, there are still many difficulties in regards to its implementation in the clinical practice [32]. A relatively high percentage of health professionals, about 70%, find it difficult to develop empathy with their health care users [32].

Age, self-reflection, appraisal, and emotions’ expressions were associated with women’s social workers empathy. Social workers had a higher score of empathy whenever they had previous work experience [55]. Additionally, there are studies that support that being female is associated with higher levels of empathy [56,57].

Research outcomes suggest that protective factors of social workers’ empathy are prosocial behavior toward work and positive personal and environmental resources [58]. Self-esteem, work engagement, and emotional regulation are also positively associated with empathy [58,59]. On the other hand, empathy is limited due to daily stress, that is a risk factor for burnout and compassion fatigue [59,60].

Empathy is positively correlated with reflective ability and emotional intelligence both in professional social workers and social work students [55,61]. According to a study in social work students in India, empathy and emotional intelligence were extracted as predictors of resilience through regression analysis. The authors underlined the need to enhance these attributes in social work students through the provision of appropriate curricular experiences [62].

The lack of empathy—or the low empathy levels—depends on several reasons. The most important are the large number of health care users that professionals have to deal with, the lack of adequate time, the focus on therapy, the predominant culture in medical schools, and the lack of training in empathy [30].

Further reasons include presumptions, a sense of superiority from the health professionals, and a fear of boundary violation. Time pressure, anxiety, a lack of self-awareness, and a lack of appropriate training, as well as the different socio-economic status, all the above do not favor empathy either [13].

According to scientific views from the Medicine field, empathy can be learned and Medical schools should educate their students in this respect [63,64]. Many studies have pointed out the necessity for future professionals to receive training in order to enhance their empathetic skills [64,65].

Although empathy is a core, quality principle for the health care professions, there are studies that show that health professionals cannot adequately express it and implement it [66,67]. According to studies in undergraduate nursing students, empirical education through learning processes can positively influence empathy [4,68]. Education is considered, both by students and professionals, as especially important for the reinforcement of empathetic skills [4,69,70].

Nevertheless, research data on the effectiveness of education in empathy are limited [71,72,73]. In a research study, conducted in the USA regarding the effect that empathy education has on health professionals, it was found that education contributes a great deal to the improvement of the therapeutic relationship [32]. In the same study, trained professionals are more likely to detect the emotion and progress of their health care users and therefore further explore and meet their needs. Education can be offered through hands-on work, multimedia use, role play, and experiential learning [32].

In a qualitative study, health professionals made suggestions regarding the enhancement of empathy. These suggestions included more holistic, educational interventions in behaviors that are central to the patient’s needs, with an emphasis on personal development, professional training, and supervision programs, rather than education in behavioral and communication skills [74].

‘Diversity Dolls’ is a hands-on educational method for the reinforcement of empathy that is used among social care students in a Greek university, so that students can instill empathetic skills in socially vulnerable populations [75]. It is believed that the use of such based-on-art methods helps social care students to feel safe, to explore, and give meaning to the real circumstances people live in, through pleasant, participatory, interactive activities [76].

Globally, creative educational methods such as journaling, art, role-play, and simulation games globally are becoming more popular in the health and social care fields helping students to increase their knowledge and skills in relation to empathy [75,76].

Teaching techniques and classroom methodologies familiarize social workers to empathetic skills [55]. In a study, among social work students, the results suggest that empathetic modeling from professors and field supervisors enhance social work students’ empathy. Social work educators should not focus on traditional teaching but they ought to concentrate on interactive and creative education that enhances the empathetic modeling and relationship between educators and students [77]. Apart from teaching social work students with mental flexibility, regulation of emotional and perspective taking, social workers should be taught empathy throughout the phenomenological psychological approach (seminars that utilize transcribed audio recordings of interactions) [78,79]. Additionally, regular supervision has a key role in enabling social workers to process their own feelings and to deal with empathy [80]. 

## 4. Conclusions

Empathy among health care users and professionals significantly contributes to how both groups behave as well as to their therapy and overall well-being. The development of empathetic skills constitutes an important priority in the education of health and social care students and should be encouraged. Educational programs should primarily be performed in a hands-on way that will strengthen the students’ personal and social skills and allow them to effectively communicate with their patients.

Moreover, health care professionals should be supported through continuous and personal development education programs as well as through supervision sessions that will allow them to develop empathetic skills. Political will is a prerequisite for the financing and encouragement of further actions.
